# CXC Chemokine‐Driven Vascular Reprogramming: Modulating Tumor Vasculature to Boost Therapeutic Response

**DOI:** 10.1002/advs.202523331

**Published:** 2026-01-20

**Authors:** Hongdan Chen, Yinde Huang, Juntong Wu, Ziying Yi, Yao Li, Zeyu Yang, Fan Zhang, Chong Li

**Affiliations:** ^1^ Department of Breast and Thyroid Surgery Chongqing General Hospital Chongqing University Chongqing China; ^2^ School of Pharmaceutical Sciences Southern Medical University Guangzhou China; ^3^ Medical Research Institute College of Pharmaceutical Sciences Southwest University Chongqing China

**Keywords:** CXC chemokine receptor, CXC chemokines, endothelial reprogramming, tumor microenvironment, vascular normalization

## Abstract

Aberrant tumor vasculature drives hypoxia, immune exclusion, and therapeutic resistance. However, current vascular normalization strategies remain primarily VEGF‐centered, relying on morphological and perfusion metrics with limited molecular readouts to monitor vessel function. This highlights the need for alternative frameworks to identify therapeutic windows and enhance vascular‐immune strategies. Here, we introduce a chemokine‐centered perspective that positions the CXC chemokine network as a dynamic regulator of vascular functionality. We highlight three core mechanistic dimensions: bidirectionality, temporal dynamics, and tissue specificity, and further emphasize the functional synergy emerging from their network‐level interactions. Building on these features, we propose the functional vascular normalization score (FVNS) and the chemokine‐guided vascular normalization window (VNW) as molecular tools for real‐time vascular assessment and therapeutic stratification. Finally, we outline CXC‐targeted strategies, including CXCR2/CXCR4 blockade, CXCL9/10/11 augmentation, and spatiotemporally controlled delivery platforms, which may extend and personalize the VNW. This chemokine‐focused paradigm provides a functional and implementable approach for the integration of vascular normalization and immune modulation in cancer therapy.

## Graphical abstract

Tumor vascular remodeling is discussed from a chemokine‐centered perspective. This review summarizes the bidirectional, temporal, and tissue‐specific roles of CXC chemokines in regulating vascular function and immune accessibility. A functional vascular normalization score is introduced as a conceptual framework to integrate dynamic vascular and immune states, informing therapeutic stratification and combination strategies.

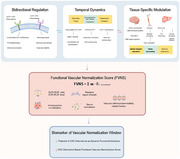

## 1 Introduction

Aberrant vasculature is a hallmark of the tumor microenvironment (TME), characterized by disorganized architecture, incomplete basement membranes, and increased vascular permeability. These abnormalities impair tissue perfusion, aggravate hypoxia and acidosis, and limit both drug delivery and immune cell infiltration [1, 2]. Anti‐angiogenic therapy, primarily targeting the vascular endothelial growth factor (VEGF) pathway, has achieved partial clinical success in solid tumors. Agents such as bevacizumab, a monoclonal antibody that neutralizes VEGF‐A, can transiently improve vascular function and enhance the efficacy of chemotherapy or immunotherapy [3, 4]. However, prolonged VEGF blockade often induces excessive vascular regression and secondary hypoxia, while tumors activate compensatory pro‐angiogenic pathways, including fibroblast growth factor (FGF), platelet‐derived growth factor (PDGF), and angiopoietin‐2 (ANGPT2), leading to therapeutic resistance [5]. Consequently, VEGF‐targeted therapy often fails to achieve sustained vascular repair or long‐term remodeling of the TME.

Recent research has therefore shifted toward multitarget tyrosine kinase inhibitors (TKIs) and interventions that regulate vascular stabilization or metabolism, such as the angiopoietin (ANGPT)/tyrosine kinase with immunoglobulin‐like and EGF‐like domains 2 (TIE2) pathway and the Notch‐delta‐like ligand 4 (DLL4) signaling axis [6, 7, 8, 9]. Although these approaches yield incremental benefits, they rely on continuous dosing and precise timing, often resulting in narrow therapeutic windows, toxicity, and adaptive resistance [10, 11, 12]. These limitations underscore the need for endogenous, self‐regulating mechanisms capable of maintaining vascular‐microenvironmental homeostasis.

Within this context, CXC chemokines, a subfamily of small secreted cytokines defined by a conserved cysteine‐X‐cysteine (CXC) motif, have emerged as key integrators of angiogenesis, vascular permeability, and immune cell trafficking [13, 14, 15, 16]. Based on the presence or absence of a Glu‐Leu‐Arg (ELR) motif, CXC chemokines are classified into ELR^+^ members (CXCL1, CXCL2, CXCL3, CXCL5, CXCL6, and CXCL8), which signal mainly through C‐X‐C chemokine receptor 1/2 (CXCR1/2) to promote angiogenesis, and ELR^−^ members (CXCL4, CXCL9, CXCL10, and CXCL11), which act via C‐X‐C chemokine receptor 3 (CXCR3) to exert angiostatic and immune‐stimulatory effects [17, 18, 19]. Collectively, accumulating evidence indicates that CXC chemokines participate in the regulation of vascular formation, remodeling, and stabilization, suggesting their potential relevance to vascular normalization.

This review presents a chemokine‐centered perspective on vascular functional reprogramming, highlighting the CXC chemokine network as a dynamic regulator of vascular‐immune interactions within the TME. By viewing vascular normalization as a process governed by context‐dependent chemokine signaling rather than static structural correction, we aim to synthesize emerging mechanistic insights, identify functional biomarkers for dynamic vascular assessment, and inform the rational design of multimodal strategies for durable tumor control.

## 2 Barriers to Sustained Functional Vascular Normalization

### 2.1 The Transient Phase of VEGF‐Induced Vascular Normalization

Anti‐angiogenic therapy targeting the VEGF/VEGFR axis induces a transient phase of vascular normalization. Moderate VEGF blockade, as achieved with bevacizumab or multitarget TKIs such as sorafenib and lenvatinib, temporarily reduces abnormal vascular sprouting and permeability, resulting in improved perfusion and oxygenation [20, 21]. However, this functional improvement is not sustained. Continued VEGF inhibition leads to excessive vessel regression and hypoxia, which stabilizes hypoxia‐inducible factor‐1α (HIF‐1α) and reactivates compensatory pro‐angiogenic pathways, including FGF, PDGF, and ANGPT2 signaling [10, 22, 23]. These adaptive responses promote alternative angiogenic programs and restore aberrant vascular growth, thereby undermining the initial benefits of VEGF inhibition.

In addition to adaptive angiogenic responses, prolonged anti‐VEGF therapy further aggravates vascular dysfunction by increasing endothelial stress and disrupting vascular integrity [24]. The resulting hypoxic and inflammatory microenvironment not only fuels tumor progression but also reinforces therapeutic resistance through metabolic reprogramming and immunosuppressive cell recruitment [24, 25, 26]. Consequently, VEGF‐centered anti‐angiogenic strategies are effective in inducing short‐term improvements in vascular function but remain insufficient to achieve durable vascular remodeling or long‐term normalization of the TME.

### 2.2 Limitations in Achieving and Maintaining Functional Vascular Normalization

Although vascular normalization is associated with improved perfusion, oxygenation, and immune accessibility [27], its assessment in both preclinical and clinical settings remains largely constrained by methodological limitations. Current evaluation strategies predominantly rely on morphological parameters, such as microvessel density (MVD), vessel diameter, and structural regularity, which provide static snapshots of vascular architecture but fail to capture dynamic changes in vascular permeability, endothelial activation, and blood flow regulation [28, 29].

Functional imaging approaches, including perfusion‐based modalities, offer indirect insights into vascular performance but are often limited by insufficient temporal resolution and delayed readouts [30]. As a result, these techniques are poorly suited to track rapid therapy‐induced fluctuations in vascular function, particularly under conditions of evolving hypoxia and inflammatory stress. Consequently, improvements detected by imaging frequently reflect downstream outcomes rather than real‐time endothelial behavior. Similarly, molecular indicators commonly used to infer vascular status, such as VEGFR2 expression, MVD, or hypoxia‐associated markers including HIF‐1α, are predominantly endpoint measurements [31]. These markers typically lag behind functional changes and are unable to resolve transient alterations in vascular integrity, immune accessibility, or endothelial‐pericyte interactions. Together, these limitations highlight that existing assessment frameworks are insufficient for dynamically monitoring vascular function or guiding adaptive therapeutic strategies. This gap underscores the need for functional, real‐time indicators that accurately reflect the evolving vascular state of the tumor microenvironment.

## 3 CXC Chemokines as Dynamic Orchestrators of Tumor Vascular Homeostasis and Immune Modulation

Tumor vasculature is regulated by signaling programs that extend beyond unidirectional angiogenic suppression and instead reflect a dynamic balance between vascular activation and stabilization. This balance is shaped by temporally and contextually regulated cues within the TME, where endothelial behavior, vascular permeability, and immune cell trafficking are tightly interconnected. Among these regulatory signals, CXC chemokines have emerged as key mediators linking vascular regulation with immune modulation (Table 1). Through ligand‐ and receptor‐specific signaling, CXC chemokines exert bidirectional effects on endothelial cells while simultaneously directing leukocyte recruitment and positioning. In contrast to VEGF‐centered pathways that primarily regulate vessel growth and density, CXC chemokine networks operate in a context‐dependent and temporally dynamic manner, enabling fine‐tuned regulation of vascular function rather than purely quantitative control of angiogenesis. By integrating angiogenic regulation with immune guidance, CXC chemokines constitute a central signaling axis governing vascular functional states in tumors, thereby providing a mechanistic foundation for subsequent discussions of bidirectionality, temporal dynamics, and tissue specificity.

**TABLE 1:** Functional spectrum of CXC chemokines in tumor vascular regulation.

ELR motif–based group	Representative chemokines	Major receptors	Predominant vascular and immune functions	Representative cancer contexts
**ELR^+^ (pro‐angiogenic / inflammatory)**	CXCL1, CXCL2	CXCR2	Promote endothelial proliferation, sprouting, and vascular permeability; recruit neutrophils and MDSCs to sustain inflammatory angiogenesis	Lung, colorectal, breast cancer
	CXCL5, CXCL6	CXCR2 (±CXCR1)	Drive chronic inflammation and pathological neovascularization; mediate CXCR2^+^ myeloid infiltration and tumor progression	Lung, liver, pancreatic cancer
	CXCL7, CXCL8 (IL‐8)	CXCR1, CXCR2	Couple platelet activation with angiogenic signaling; enhance vascular leakage and endothelial activation	Breast, ovarian, colorectal cancer
	CXCL15 *(mouse‐specific, CXCL8‐like)*	CXCR2	Functional homolog of human CXCL8; mediates neutrophil recruitment and angiogenic inflammation in murine tumor models	Murine models of lung and colon cancer
**ELR^−^ (angiostatic / immune‐activating)**	CXCL9, CXCL10, CXCL11	CXCR3	Inhibit aberrant angiogenesis; promote recruitment of CD8^+^ T cells and NK cells; contribute to vascular normalization and immune activation	Melanoma, NSCLC, hepatocellular carcinoma
**ELR^−^ (metastasis‐associated / endothelial‐tumor crosstalk)**	CXCL12	CXCR4, CXCR7	Regulate endothelial–tumor interactions, cell migration, and pre‐metastatic niche formation; remodel vasculature during metastasis	Breast, liver, brain metastasis
**ELR^−^ (stromal / immune‐organizing)**	CXCL13	CXCR5	Induce tertiary lymphoid structure (TLS) formation; facilitate B‐cell and Th‐cell infiltration; shape adaptive immune niches	Breast cancer, lymphoma
	CXCL14	Unknown	Exert tumor‐suppressive or immune‐recruiting activity; maintain stromal–endothelial homeostasis	Breast, prostate, head and neck cancer
**ELR^−^ (dual‐functional / context‐dependent chemokines)**	CXCL16, CXCL17	CXCR6, GPR35 (putative)	Regulate immune‐cell trafficking and vascular remodeling; exhibit either pro‐angiogenic or angiostatic activity depending on tumor type and immune context	Lung, liver, colorectal cancer

### 3.1 Bidirectional Regulation: Opposing Vascular Programs Mediated by ELR^+^ and ELR^−^ CXC Chemokines

ELR^+^ chemokines (CXCL1/2/3/5/6/8) primarily signal through CXCR1/2, activating MAPK/ERK and PI3K/AKT cascades to promote endothelial proliferation, migration, and sprouting, thereby supporting pro‐angiogenic vascular programs [17, 32, 33]. In contrast, ELR^−^ chemokines (CXCL4/9/10/11) engage CXCR3 isoforms to modulate endothelial adhesion and motility, and are generally associated with angiogenic restraint and context‐dependent stabilization‐related features [34, 35].

**FIGURE 1:**
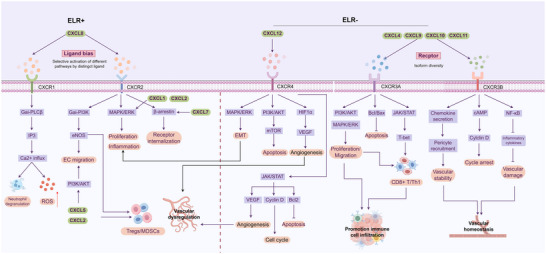
Differential signaling and vascular effects of ELR^+^ and ELR^−^ CXC chemokines. ELR^+^ CXC chemokines (e.g., CXCL1, CXCL2, CXCL5, CXCL8) predominantly bind to CXCR1/2 and activate downstream pathways such as MAPK/ERK, PI3K/AKT, and β‐arrestin signaling, which are commonly associated with endothelial proliferation, migration, and angiogenesis, and pro‐inflammatory signaling, as well as recruitment of immunosuppressive cells. By contrast, ELR^−^ CXC chemokines (e.g., CXCL4, CXCL9, CXCL10, CXCL11) mainly signal through CXCR3 isoforms, regulating endothelial behavior, angiostatic activity, and immune cell trafficking, thereby exerting anti‐angiogenic and immunomodulatory effects. CXCL12, via CXCR4/7, activates PI3K/AKT‐, mTOR‐, and HIF1α‐VEGF‐associated pathways to support angiogenesis and cell survival, yet also contributes to tumor progression and metastasis. Together, the interplay of ligand bias, receptor diversity, and downstream signaling cascades highlights the context‐dependent roles of the CXC family in vascular dysregulation, immune remodeling, and tumor vascular homeostasis.

The functional output of CXC signaling is highly context‐dependent, and is shaped by ligand expression levels, microenvironmental conditions, receptor isoform usage, and tumor stage. Even ligands sharing the same receptor can produce distinct outcomes: both CXCL8 and CXCL5 activate CXCR2, yet CXCL8 preferentially promotes cytoskeletal remodeling and vascular permeability via ERK/AKT signaling, whereas CXCL5 tends to recruit neutrophils through p38‐NF‐κB signaling [28, 29]. Similarly, CXCR3‐A mediates Gαi‐PI3K‐driven chemotaxis, while CXCR3‐B triggers cAMP/PKA‐p38‐caspase pathways that constrain endothelial growth [36, 37]. Such ligand bias and receptor isoform diversity endow the CXC network with bidirectional and plastic regulatory capacity over vascular phenotype (Figure 1). Nevertheless, current research remains focused on isolated ligand‐receptor pairs, and network‐level regulatory dynamics, especially in vivo isoform switching, are still insufficiently characterized, limiting a comprehensive understanding of CXC‐mediated vascular modulation.

### 3.2 Temporal Dynamics: Stage‐Associated Patterns of Pro‐Angiogenic and Angiostatic Chemokine Signaling

**FIGURE 2:**
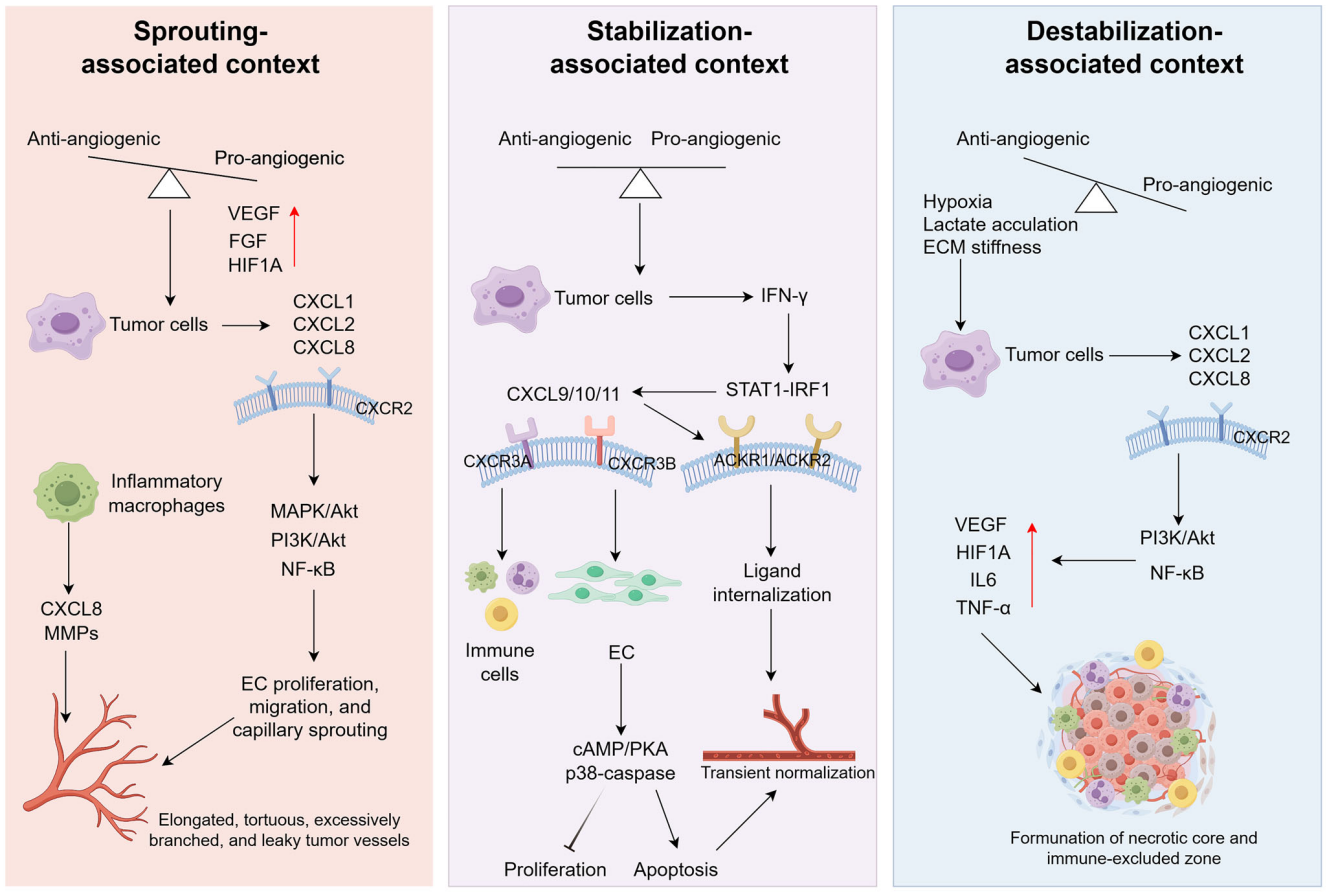
Temporal dynamics of CXC chemokine signaling in tumor vascular regulation. CXC chemokines exhibit stage‐dependent roles during tumor vascular remodeling. In a sprouting‐associated context, hypoxia‐ and inflammation‐induced CXCL1/2/8 predominantly engage CXCR2 signaling, supporting endothelial activation, proliferation, and capillary sprouting. A stabilization‐associated context has been reported in which IFN‐γ‐STAT1‐IRF1‐driven CXCL9/10/11 signaling, acting mainly through CXCR3‐B and atypical chemokine receptors, coincides with reduced endothelial proliferation and features compatible with transient vascular stabilization. In a destabilization‐associated context, sustained hypoxia, lactate accumulation, and increased matrix stiffness are associated with renewed ELR^+^ chemokine dominance (notably CXCL8), re‐engagement of CXCR2‐PI3K/AKT‐NF‐κB signaling, and induction of VEGF and inflammatory mediators, collectively promoting vascular hyperpermeability, collapse, and immune exclusion.

CXC chemokines exhibit distinct temporal phases during tumor progression and therapeutic intervention (Figure 2). Hypoxia‐driven ELR^+^ signaling is commonly associated with sprouting angiogenesis, while IFN‐γ‐induced CXCL9/10/11 expression has been reported in certain therapeutic contexts and has been associated with angiogenic restraint and endothelial functional modulation in specific therapeutic contexts, features that have been discussed in relation to a putative vascular normalization‐like state. As hypoxia deepens and extracellular matrix stiffening progresses, ELR^+^ chemokine dominance often re‐emerges, contributing to vascular destabilization.

In sprouting‐associated settings, hypoxia and inflammation activate HIF‐1α, NF‐κB and AP‐1, inducing CXCL1/2/8 expression to drive endothelial proliferation and migration through CXCR2‐MAPK/ERK and PI3K/AKT signaling [38, 39]. Macrophage‐derived CXCL8 and matrix‐remodeling enzymes further enhance endothelial activation and extracellular matrix degradation [40, 41]. Under anti‐angiogenic or immune‐based interventions, a transient reconfiguration of the chemokine milieu may occur, accompanied by partial restoration of vascular perfusion, a state compatible with a functional normalization‐like phase [42, 43]. During this phase, IFN‐γ‐STAT1‐IRF1‐driven CXCL9/10/11 upregulation activates CXCR3‐B‐cAMP/PKA‐p38‐caspase signaling, limiting endothelial proliferation and promoting apoptosis [44, 45, 46], while in certain in vivo contexts this is associated with increased pericyte coverage and vascular features consistent with partial stabilization [34]. However, competition between CXCR3‐A and CXCR3‐B signaling and chemokine scavenging by ACKR1/2 restrict the duration of this stabilization phase [47, 48, 49]. As hypoxia, lactate accumulation, and matrix rigidity increase, ELR^+^ chemokines such as CXCL8 are re‐induced, re‐activating CXCR2‐PI3K/AKT‐NF‐κB pathways to stimulate VEGF, IL‐6 and TNF‐α expression [50, 51, 52], ultimately leading to excessive sprouting, vascular leakage, and loss of vascular homeostasis.

### 3.3 Tissue‐Specific Modulation: Vascular‐Immune Interactions Mediated by CXC Chemokines

**FIGURE 3:**
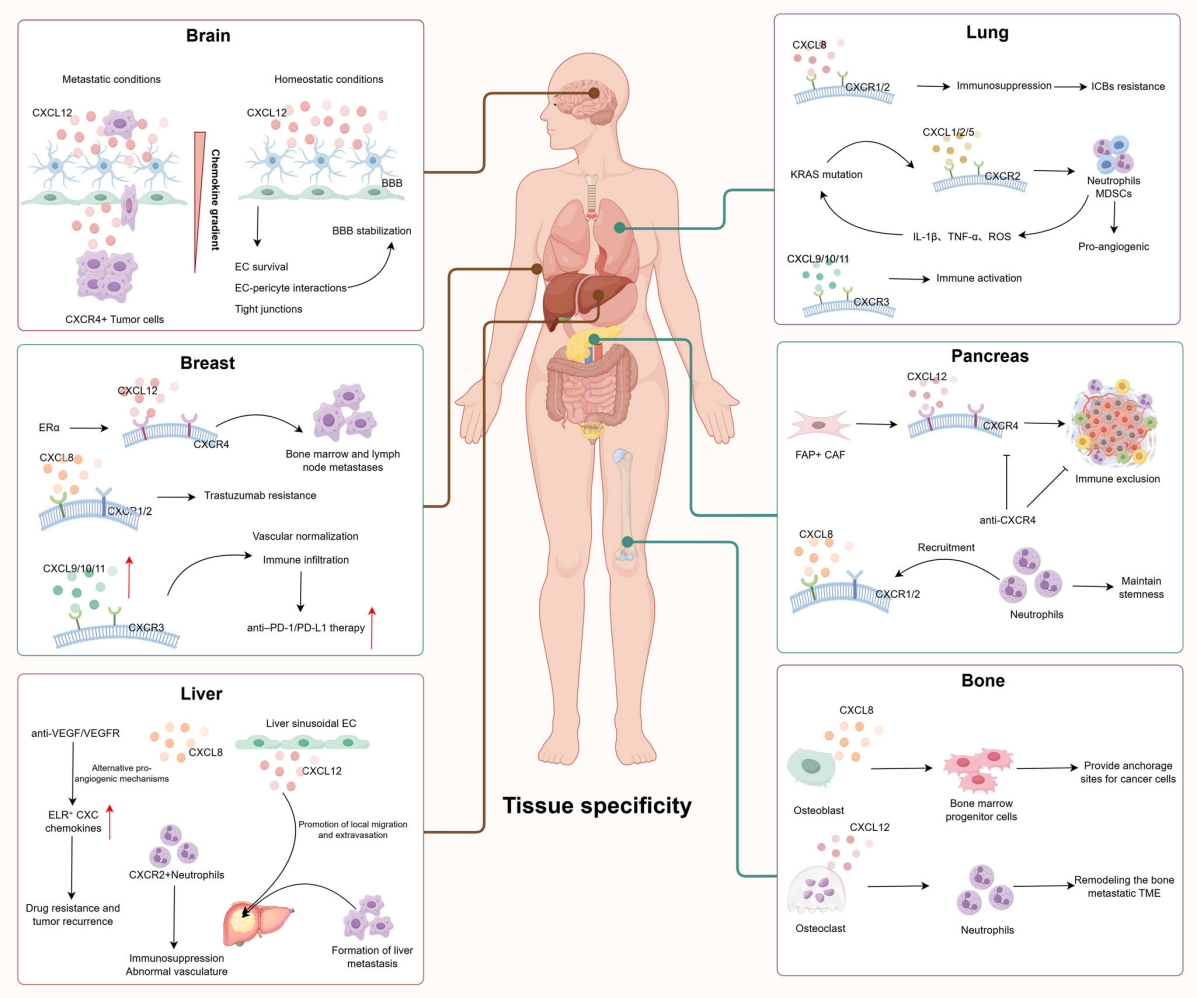
Tissue‐specific roles of CXC chemokines in tumor vascular and immune regulation. CXC chemokines display pronounced tissue specificity that is shaped by vascular structure, metabolic context, and immune milieu. In the brain, CXCL12‐CXCR4 gradients have been implicated in facilitating tumor cell interactions with the blood‐brain barrier, while under steady‐state conditions CXCL12 contributes to barrier integrity. In the lung, CXCL1/2/5‐CXCR2 signaling drives pro‐angiogenic and immunosuppressive responses downstream of KRAS mutations, whereas CXCL9/10/11‐CXCR3 supports immune cell infiltration. In the liver, compensatory induction of CXCL1/8 and CXCL12 following anti‐VEGF therapy has been linked to neutrophil recruitment, immunosuppression, and metastasis progression. In breast, pancreas, and bone, CXCL12‐CXCR4 and CXCL8 signaling contribute to therapy resistance, immune exclusion, and metastatic niche remodeling. This organ‐dependent heterogeneity highlights the vascular‐immune interface as a key determinant of organ‐specific tumor progression and therapeutic response.

CXC chemokines exhibit organ‐specific functional heterogeneity, shaped by vascular architecture, metabolic state, and the local immune milieu (Figure 3). In brain metastasis, the CXCL12‐CXCR4 axis forms chemotactic gradients at the blood–brain barrier, promoting tumor cell adhesion and migration [53], while PI3K/AKT‐mediated junctional regulation influences barrier integrity and metastatic dissemination [54]. Under physiological conditions, this pathway contributes to the maintenance of vascular stability [55], demonstrating context‐dependent duality. In the bone marrow niche, CXCL8 promotes angiogenesis and RANKL‐dependent osteoclastogenesis, reorganizing hematopoietic and metabolic environments to support tumor colonization [56]. In liver tumors, anti‐VEGF therapy induces compensatory activation of the CXCL1/2‐CXCR2 axis [57], recruiting neutrophils and sustaining pro‐angiogenic inflammation [58], whereas IFN‐γ‐induced CXCL9/10 expression in sinusoidal endothelium modulates vascular remodeling and immune cell infiltration [59, 60]. Thus, CXC‐mediated vascular effects differ across tissues, ranging from barrier regulation in the brain to metabolic and osteogenic remodeling in bone and immune adaptation in the liver, highlighting the need to interpret CXC activity within organ‐specific vascular‐immune microecologies.

However, most studies remain single‐tissue focused; integrating spatial transcriptomics with functional vascular imaging may help define cross‐organ patterns of CXC regulation and inform the development of organ‐tailored strategies for modulating vascular function.

### 3.4 Network Synergy: Integrative CXC Chemokine Circuits Connecting Vasculature, Immunity, and Stroma

**FIGURE 4:**
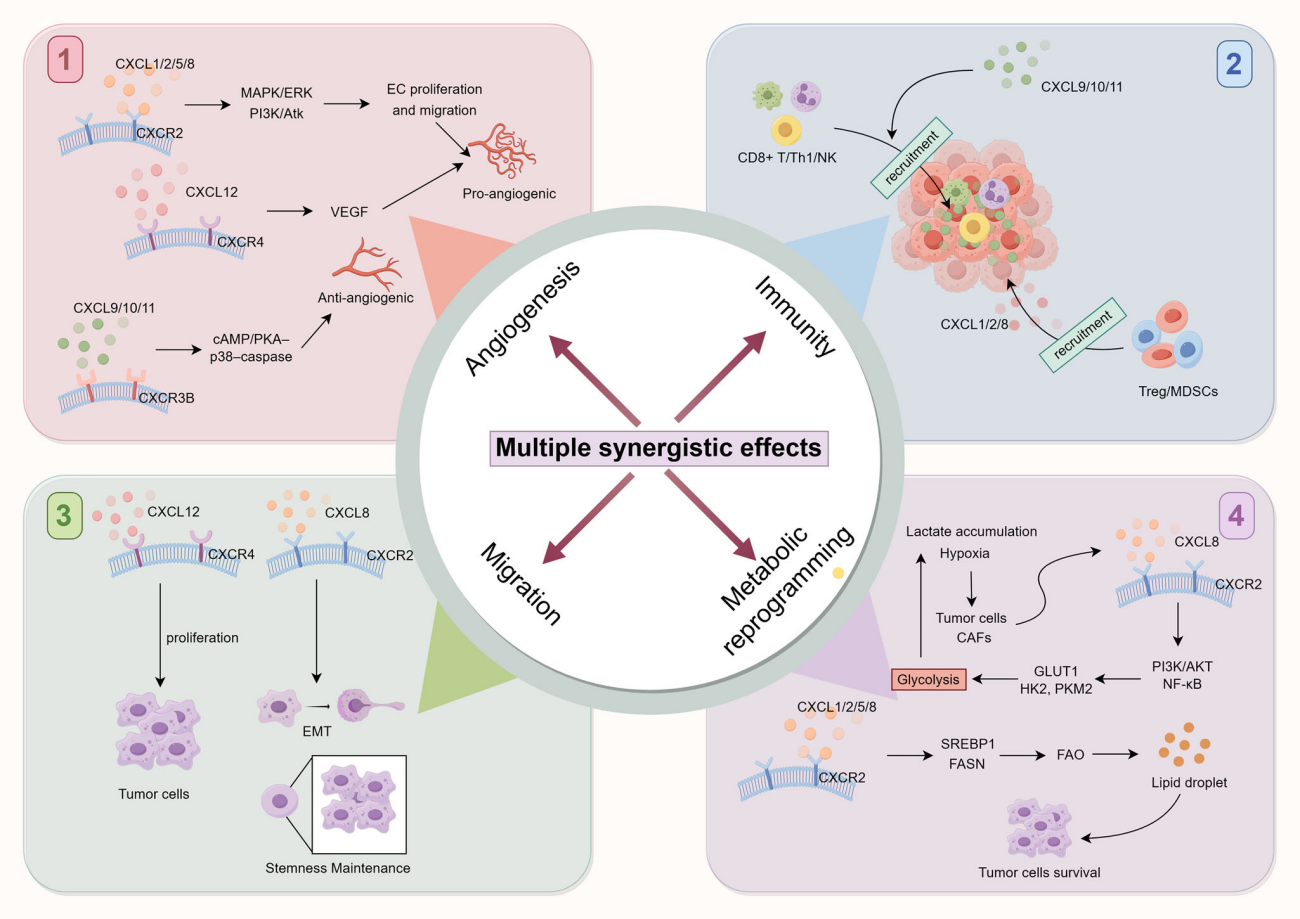
Multiple synergistic effects of CXC chemokines in tumor progression. CXC chemokines coordinate a network of interrelated process within the TME. Through CXCR2 and CXCR4 signaling, ELR^+^ ligands such as CXCL1/2/5/8 and CXCL12 stimulate endothelial activation, motility and VEGF‐dependent angiogenic programs, whereas ELR^−^ ligands (CXCL9/10/11) acting via CXCR3‐B mediate anti‐angiogenic signaling. In the immune compartment, CXCL9/10/11 preferentially guide effector CD8^+^ T cells, Th1 cells, and NK cells, while CXCL1/2/8 bias immune composition toward Tregs and myeloid‐derived suppressor cells (MDSCs), thereby establishing divergent immune contexts. CXCL12‐CXCR4 and CXCL8‐CXCR2 signaling reinforce tumor cell proliferation, epithelial‐mesenchymal transition, and stem‐like traits. In addition, hypoxia‐ and lactate‐induced CXCL8 expression links metabolic stress to adaptive signaling through PI3K/AKT‐NF‐κB and lipid metabolic pathways, enhancing tumor cell survival. These axes illustrate the integrated roles of CXC chemokines in coupling angiogenesis, immunity, migration, and metabolic adaptation.

CXC chemokines function through interconnected signaling circuits, rather than isolated ligand‐receptor events. The CXCL8‐CXCR2 axis not only promotes endothelial proliferation and granulocyte recruitment, but also supports cancer stemness, linking angiogenesis, immune suppression, and therapeutic resistance [61]. The CXCL12‐CXCR4 axis regulates cell migration and endothelial/hematopoietic survival, together with CXCL1/2, contributes to pre‐metastatic niche formation [33, 62]. Conversely, IFN‐γ–induced CXCL9/10/11 activate CXCR3‐B to promote vascular inhibition and immune activation [63, 64], while CXCR3‐A may enhance tumor motility [65], demonstrating receptor‐dependent functional duality. At metabolic and stromal levels, hypoxia and lactate enhance CXCL8, and ECM remodeling releases matrix‐bound chemokines, thereby extending signaling duration [66, 67]. Together, these interactions form a network integrating vascular, immune, and stromal states, in which cooperative signaling outweighs the effects of single signaling axis (Figure 4). Accordingly, interpreting CXC function in tumors requires a network‐level perspective that considers coordinated signaling dynamics rather than isolated ligand–receptor activities.

## 4 CXC‐Mediated Identification of the Vascular Normalization Window

Vascular normalization has long been recognized as a critical but transient phase during anti‐angiogenic therapy, in which improvements in perfusion, oxygenation, and immune accessibility may enhance combination efficacy. However, despite its conceptual importance, the clinical translation of the vascular normalization window (VNW) remains limited by the lack of systematic and dynamic criteria to define when this functional state arises and declines. Clinical observations following bevacizumab‐ treatment illustrate this challenge: transient gains in perfusion and immune infiltration can be detected within days, yet excessive vessel pruning rapidly induces hypoxia, vascular dysfunction, and immune escape, rendering therapeutic timing highly vulnerable to misalignment [68, 69]. A fundamental limitation underlying this challenge is that current approaches primarily assess vascular recovery using static or outcome‐oriented parameters. Conventional imaging and perfusion metrics largely reflect structural changes and lack the temporal resolution required to capture dynamic vascular function [70]. Similarly, molecular markers such as VEGFR2 expression, MVD, and HIF‐1α accumulation represent delayed or endpoint indicators that fail to resolve rapid transitions in vascular state [71]. Collectively, these limitations suggest that the VNW is not simply difficult to exploit, but may be inadequately characterized by existing structural and static evaluation frameworks, underscoring the need to reframe vascular normalization as a dynamic functional state rather than a fixed temporal window.

### 4.1 Potential of CXC Chemokines as Dynamic Functional Indicators

Identifying molecular signals that dynamically reflect vascular function remains a major challenge. Among CXC‐associated pathways, chemokines linked to hypoxia‐driven inflammation and interferon‐mediated immune activation have been most frequently explored as candidate indicators of vascular functional states. Chemokines such as CXCL1, CXCL2, and CXCL8 are consistently associated with inflammatory angiogenesis, vascular hyperpermeability, and tissue stress responses [72, 73]. While this sensitivity renders them effective markers of pathological angiogenic activation, their expression is strongly influenced by acute inflammation and tissue damage, limiting their specificity for improvements in functional vascular function.

In contrast, interferon‐responsive pathways centered on CXCL9 and CXCL10 have shown more consistent associations with therapy‐induced vascular and immune changes. Across preclinical models and clinical cohorts, CXCL9 and CXCL10 expression correlates with CD8^+^ T‐cell recruitment and improved vascular perfusion during effective treatment [74, 75]. Notably, longitudinal analyses during anti‐PD‐1 therapy indicate that temporal fluctuations, rather than baseline abundance, of CXCL10 and CXCL11 are associated with therapeutic response and survival [76]. However, these signals remain tightly coupled to immune activation, complicating discrimination between intrinsic modulation of vascular function and immune‐driven effects. Collectively, current evidence supports interpreting CXC chemokine patterns as context‐dependent, dynamic indicators of vascular functional states, rather than definitive or unidirectional biomarkers.

### 4.2 CXC Chemokine‐Based Functional Vascular Normalization Score

**FIGURE 5:**
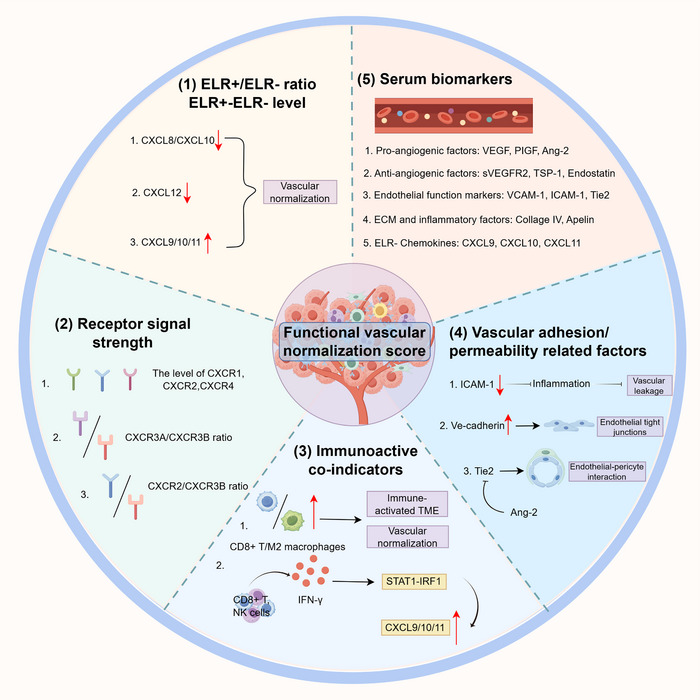
Components of a functional vascular normalization score (FVNS). The FVNS conceptually integrates multiple functional dimensions to capture dynamic vascular states in tumors. Key determinants include the relative balance and abundance of ELR^+^ versus ELR^−^ chemokines (e.g., CXCL8/CXCL10, CXCL12, CXCL9/10/11) and the activity of their corresponding receptors, such as CXCR1/2, CXCR3A/B, and CXCR4. Immune‐associated indicators, including CD8^+^ T cell and macrophage functional states, IFN‐γ‐STAT1‐IRF1 signaling, and CXCL9/10/11 induction, serve as readouts of vascular–immune crosstalk. Vascular adhesion and permeability markers (e.g., ICAM‐1, VE‐cadherin, Tie2) inform endothelial integrity and vessel stabilization, including pericyte coverage. In parallel, circulating biomarkers encompassing pro‐ and anti‐angiogenic factors, endothelial function markers, ECM components, and ELR chemokines provide accessible, systemic correlates. Together, these variables are integrated here as a composite framework for evaluating vascular normalization in both experimental and clinical settings.

The clinical translation of vascular normalization is limited by the lack of an integrative framework capable of capturing dynamic vascular functional states. To address this limitation, we propose a functional vascular normalization score (FVNS) as a conceptual, semi‐quantitative index designed to estimate the degree of vascular functional modulation at a given time point (Figure 5).

FVNS is conceived as a composite framework integrating multiple chemokine‐associated functional modules that reflect key aspects of vascular regulation. These include angiogenic balance, represented by the relative dominance of ELR^+^ versus ELR^−^ CXC chemokines (e.g., CXCL8/CXCL10) [30]; endothelial functional state, reflected by CXCR2‐ versus CXCR3‐associated signaling programs [31]; immune‐vascular coupling, captured by immune activation parameters such as CD8^+^ T‐cell infiltration and IFN‐γ signaling [77, 78]; and endothelial barrier integrity, indicated by markers related to junctional stability and permeability, including ICAM‐1, VE‐cadherin, and Ang‐2/Tie2 signaling [78, 79]. In addition, circulating vascular‐associated factors may further support non‐invasive monitoring of these dynamics [80]. Here, dynamic monitoring is primarily envisioned through longitudinal blood‐based analyses, given their feasibility for repeated assessment over the course of therapy, with tissue biopsies used selectively to provide intratumoral context.

$$\mathrm{Conceptually},\;\mathrm{FVNS}\;\mathrm{can}\;\mathrm{be}\;\mathrm{expressed}\;\mathrm{as}:\mathrm{FVNS}=\Sigma w_{i}\cdot F_{i}$$

where F_i_ denotes normalized functional dimensions and w_i_ represents context‐dependent weighting factors reflecting their relative contribution under specific tumor and therapeutic settings. For instance, in immune‐excluded or severely hypoxic tumors, vascular dysfunction is largely driven by angiogenic imbalance and endothelial barrier disruption [81], suggesting that ELR^+^/ELR^−^ chemokine dominance or barrier‐associated parameters may carry greater interpretive weight, whereas immune‐associated features are less informative at this stage. Conversely, in immune‐inflamed or therapy‐responsive contexts, where vascular accessibility and immune trafficking become functionally coupled [82, 83], indicators such as CXCL9/10‐associated signaling, CD8^+^ T‐cell infiltration, or IFN‐γ activity are more likely to reflect shifts toward functional normalization. Similarly, during early phases of anti‐angiogenic intervention, changes in endothelial signaling or barrier integrity may precede immune engagement [84], whereas during later combination regimens, immune accessibility may offer a more sensitive readout of a functional normalization window [85, 86]. Importantly, these considerations represent heuristic, mechanism‐informed interpretations rather than validated hierarchies, underscoring FVNS as a conceptual framework for reasoning about dynamic vascular states rather than a quantitative scoring system. The summation reflects the additive contribution of partially independent functional dimensions rather than a deterministic algorithm. Existing approaches to assess vascular normalization typically rely on structural metrics such as MVD or vessel morphology, functional imaging modalities that capture perfusion or hypoxia at defined endpoints [87, 88], or single molecular markers including VEGF or HIF‐1α [24]. While informative, these measures are often static, delayed, or insufficient to integrate immune‐vascular interactions. In contrast, FVNS is designed to integrate angiogenic balance, endothelial functional state, and immune accessibility into a unified, functional framework, thereby capturing dynamic vascular states beyond individual parameters. Accordingly, FVNS is not proposed as a finalized clinical tool but as a hypothesis‐generating conceptual framework to describe vascular regulation as a dynamic, regulatable functional process.

## 5 The Therapeutic Effects and Application of CXC Chemokines

Beyond serving as biomarkers for vascular status, CXC chemokines have the potential to regulate vascular function, particularly in cancer therapy. By modulating specific CXC chemokine pathways, they can promote the restoration or modulation of vascular function. This makes CXC chemokines key not only in evaluating therapeutic responses but also as potential targets for next‐generation combination therapies. In this shift, the CXCL12‐CXCR4 and CXCL9/10/11‐CXCR3 axes have emerged as candidate regulators of vascular functional states. With the clinical advancement of small molecules, antibodies, and fusion proteins, CXC chemokines offer new adjunctive strategies for combination cancer therapy [89].

### 5.1 Receptor Antagonists

In the development of therapeutics targeting CXC chemokine signaling, receptor antagonists represent one of the most clinically actionable strategies to date. Among them, CXCR4 and CXCR2 have emerged as pivotal nodes in distinct but converging pathways: the CXCL12–CXCR4 axis orchestrates angiogenesis and immune evasion, whereas the CXCL8/CXCL1–CXCR2 network links inflammatory chemokine signaling to tumor‐associated vascular dysfunction (Table 2).

#### 5.1.1 CXCR4 Antagonists

CXCR4 antagonists are the most clinically advanced agents targeting CXC chemokine signaling. CXCR4, a G protein–coupled receptor for CXCL12, is highly expressed in diverse tumors, driving cell migration, angiogenesis, and immune evasion [90]. The CXCL12‐CXCR4 axis mediates compensatory angiogenesis and immune exclusion during anti‐VEGF therapy [91]. The prototype inhibitor AMD3100 (plerixafor) suppresses aberrant vascular sprouting, improves perfusion, and enhances T‐cell infiltration by blocking CXCL12‐induced endothelial migration and matrix metalloproteinase (MMP) activation [92, 93, 94]. In preclinical models, combining CXCR4 blockade with immune checkpoint or anti‐VEGF therapy synergistically restrains tumor growth and alleviates immunosuppression [95, 96, 97]. Early‐phase trials support these findings, showing improved immune activation and delayed progression in solid tumors [98, 99]. However, as CXCL12‐CXCR4 also maintains hematopoietic and vascular homeostasis, prolonged inhibition may cause bone marrow suppression or cardiovascular toxicity [100]. Thus, precise temporal scheduling and dosage optimization remain key for translation. Taken together, CXCR4 antagonists have established a relatively well‐defined developmental trajectory, serving as representative modulators of vascular function and offering a useful translational paradigm for other CXC‐related pathways.

#### 5.1.2 CXCR2 Antagonists

CXCR2 serves as the principal receptor for ELR^+^ CXC chemokines such as CXCL1, CXCL2, CXCL5, and CXCL8, playing a central role in inflammation‐driven angiogenesis and immune suppression [101]. Pharmacologic inhibition of CXCR2 effectively blocks the recruitment of neutrophils and MDSCs, thereby limiting VEGF‐independent neovascularization and facilitating immune cell infiltration into tumors [31, 102]. Representative agents include Reparixin, a dual CXCR1/2 inhibitor, which in a phase II breast cancer trial showed delayed disease progression when combined with chemotherapy [103], and AZD5069, a selective CXCR2 antagonist currently under clinical investigation in prostate cancer [104]. Furthermore, preclinical studies have shown that CXCR2 inhibition reprograms the immunosuppressive microenvironment and restores sensitivity to PD‐1/PD‐L1 blockade, thereby enhancing antitumor efficacy and reducing metastatic dissemination [105]. However, excessive inhibition of CXCR2 may also compromise host antimicrobial defense mechanisms due to impaired neutrophil trafficking [106]. Given the marked variability of CXCL8 expression across tumor types and disease stages, the therapeutic success of CXCR2 antagonists in immunotherapy will rely on precise patient stratification and rational combination strategies to balance efficacy and safety.

### 5.2 Immunomodulatory Adjuvants

#### 5.2.1 Cell Engineering

Beyond receptor antagonism, amplifying the activity of ELR^−^ type CXC chemokines, particularly the CXCL9/10/11‐CXCR3 axis, represents a strategy to integrate angiogenic restraint and immune activation, with potential implications for vascular functional modulation. This signaling pathway not only suppresses aberrant angiogenesis but also establishes a robust chemotactic gradient that directs the infiltration of CD8^+^ T cells, NK cells, and Th1 cells into tumors [107, 108], thereby bridging improved vascular function and enhanced antitumor immunity. Preclinical studies have demonstrated that localized delivery of CXCL9 can markedly enhance intratumoral immune cell infiltration and sensitize tumors to immune checkpoint blockade (ICB), without inducing overt systemic toxicity [109]. Engineered dendritic cells or macrophages programmed to sustain CXCL9/10 secretion have been applied via intratumoral administration, resulting in markedly improved responsiveness to ICB [110]. Therefore, combining anti‐VEGF therapy with localized CXCL10 delivery may extend vascular functional states, synchronizing perfusion and immune infiltration via CXCR3 signaling [111]. Clinical correlative studies have shown that elevated CXCL9/CXCL10 expression is frequently associated with improved progression‐free survival, overall survival, or response to immune checkpoint inhibitors [112, 113], suggesting that these chemokines may serve not only as therapeutic targets but also as potential predictive biomarkers.

#### 5.2.2 Oncolytic Viruses

Another promising approach involves employing oncolytic viruses as delivery platforms for CXCL chemokines. Beyond inducing tumor lysis and antigen release, oncolytic viruses engineered to express CXCL11 can further amplify effector T‐cell recruitment [114]. For instance, CXCL11‐expressing vaccinia viruses markedly increased CD8^+^ T‐cell infiltration and prolonged survival in colorectal cancer and mesothelioma models [115, 116]. Similarly, adenoviruses encoding CXCL11 enhanced T‐cell persistence and improved glioblastoma clearance when combined with CAR‐T therapy [114]. In addition, local treatments such as radiotherapy can upregulate endogenous CXCL10, forming a positive feedback loop with exogenous delivery [117]. However, the heterogeneous expression of CXCL9/10/11 and the functional divergence of CXCR3 isoforms indicate that indiscriminate amplification may not consistently yield therapeutic benefit. Future strategies should emphasize temporally controlled and spatially targeted activation of CXCL signaling, integrating it with anti‐VEGF, ICB, radiotherapy, or oncolytic virus regimens, and guided by dynamic biomarkers to achieve coordinated synergy between vascular repair and immune activation.

#### 5.2.3 Fusion Proteins

In contrast to cell‐based engineering and viral delivery platforms, fusion protein‐based strategies aim to enhance the stability, bioavailability, and controllability of chemokine signaling while avoiding the complexity of living or replicating systems. Conjugating CXC chemokines with antibodies or functional fragments provides a balance between prolonged half‐life and target specificity. For example, a DPP‐4‐resistant CXCL10‐IgG Fc fusion protein has been shown to extend in vivo persistence while retaining CXCR3‐binding activity, thereby enhancing CD8^+^ T‐cell infiltration and potentiating anti‐PD‐1 efficacy in melanoma models. [118]. This strategy parallels established immune‐cytokine approaches, such as L19‐IL‐2 and L19‐TNF, which improve cytokine localization and therapeutic index. Building on this paradigm, fusion protein engineering has further evolved toward dual‐cytokine constructs that activate multiple immune pathways in parallel [119]. these findings position fusion proteins as modular immunomodulatory adjuvants for integrating CXC chemokine signaling into next‐generation immunotherapy.

#### 5.2.4 Therapeutic Antibodies

Antibody‐based therapies are emerging as biological complements to small‐molecule CXC inhibitors, offering higher specificity and sustained activity. The anti‐CXCL8 antibody BMS‐986253 (HuMax‐IL8) neutralizes IL‐8‐driven angiogenesis and myeloid recruitment [120]. Early clinical evaluation of BMS‐986253 (HuMax‐IL8) in combination with nivolumab (NCT03689699) demonstrated manageable safety and indications of on‐target activity, including reductions in circulating IL‐8 and enhanced immune cell activation. Antibodies against CXCR4, such as Ulocuplumab (BMS‐936564), have demonstrated stromal disruption and preliminary efficacy in leukemia [121], while preclinical models reveal associated improvements in vascular behavior and immune infiltration. Collectively, these biologics exemplify the translational maturation of CXC‐targeted therapy, suggesting that ligand neutralization and receptor blockade can jointly reprogram tumor vasculature and potentiate immunotherapy.

### 5.3 Smart Drug‐Delivery Systems

The clinical translation of CXC chemokines has long been hindered by their short half‐life, diffuse biodistribution, and potential systemic toxicity. Enhancing molecular stability and targeting specificity, while achieving spatiotemporally controlled release, remains a major pharmacological challenge. Recent advances in fusion protein design and diverse nano‐delivery platforms have provided viable solutions to these limitations, improving pharmacokinetic profiles and establishing a technological foundation for coordinated modulation of vascular repair and immune activation.

Compared with molecular stabilization achieved by fusion protein engineering, delivery systems place greater emphasis on spatiotemporal control of chemokine release. Through rational carrier design, they not only prolong in vivo retention but also enable context‐dependent delivery tailored to the complexity of the TME. Depending on the implementation approach, current delivery strategies can be broadly categorized as follows:

1. *Conventional Carrier‐Based Delivery* This approach primarily relies on established platforms such as liposomes, polymeric nanoparticles, and hydrogels. These carriers can encapsulate CXCL9/10 proteins or their mRNAs to generate high local concentrations within tumor lesions while minimizing systemic toxicity. Local release of CXCL10‐loaded liposomes has been shown to induce macrophage polarization toward an M1‐like phenotype, increase the infiltration of tumor‐resident natural killer cells, and synergize with ICB [122]. Although these systems benefit from well‐established fabrication processes, they generally suffer from limited release precision and a narrow therapeutic window.
2. *Stimuli‐Responsive and Lesion‐Activated Systems* These systems improve the specificity of chemokine delivery, as CXCL8‐responsive microspheres release payloads selectively in inflamed regions, minimizing off‐target toxicity [123]. Likewise, CXCL12‐shielding nanocarriers ameliorate vascular abnormalities and alleviate immunosuppression by blocking the CXCL12‐CXCR4 signaling [124, 125].Recent advances in pancreas‐targeted IL‐12 mRNA lipid nanoparticles have demonstrated potent reprogramming of the tumor immune microenvironment [126]. An optimized strategy could further leverage regions of elevated CXCL10 expression as endogenous triggers for selective IL‐12 release, thereby synchronizing vascular stabilization with immune activation. Such strategies emphasize demand release, offering new avenues to address the challenges of short therapeutic windows and high tumor heterogeneity.
3. *Emerging Genetic and Chemical Engineering Platforms* These approaches greatly expand the scope of chemokine delivery. Layer‐by‐layer nanoparticles (LbL‐NPs) efficiently encapsulate IL‐12 for targeted release, markedly reducing systemic toxicity [127, 128]. Programmable mRNA systems can trigger IL‐12 expression within the TME, while engineered stem cells deliver cytokines such as IFN‐β directly to lesions [129]. Chemical modifications, particularly PEGylation, further enhance cytokine stability; notably, Bempegaldesleukin (NKTR‐214), a PEGylated IL‐2 variant that preferentially activates CD122 signaling to boost CD8^+^ T‐ and NK‐cell function [130], has advanced into clinical trials, demonstrating translational promise [131].

**TABLE 2:** Representative strategies modulating the CXC chemokine axis.

Strategy category	Representative agents / tools	Mechanistic rationale	Clinical status
**CXCR4 antagonists**	AMD3100 (plerixafor); BKT‐140	Interrupts CXCL12‐CXCR4 signaling, which can lessen endothelial migration/leakage and immune exclusion	NCT04177810, PhaseII, Pancreatic Cancer; NCT02179970, PhaseI, Pancreatic, Ovarian and Colorectal Cancers; NCT01010880, PhaseI/II, Multiple Myeloma.
**CXCR2 antagonists**	Reparixin (CXCR1/2); AZD5069 (CXCR2)	Limits ELR^+^ CXC–driven inflammatory angiogenesis and myeloid recruitment, potentially improving immune ingress	NCT01861054, PhaseII, Breast Cancer; NCT02370238, PhaseII, Breast Cancer; NCT02001974, PhaseI, Breast Cancer. NCT03177187, PhaseI/II, Prostate Cancer; NCT02499328, PhaseI/II, Solid Tumors.
**CXCR4/CXCR7 axis‐dual/next‐gen blockers**	Motixafortide/BL‐8040; Mavorixafor (X4P‐001)	Disrupts CXCL12‐dependent trafficking and stromal crosstalk, potentially enhancing immune access	NCT02826486, PhaseII, Pancreatic Cancer. NCT02667886, PhaseI/II, Renal Cell Carcinoma.
**Oncolytic virus (OV) vectors**	CXCL9/10/11‐expressing oncolytic adenoviruses; CXCL11‐armed vaccinia virus	Intratumoral OV may raise chemokine/alarmin signals and recruit effector T cells, augmenting ICI activity	Preclinical stage
**CXCL9/10/11 augmentation**	Recombinant/mRNA delivery; engineered cells/viral payloads	Elevates CXCR3^+^ T/NK‐cell trafficking and endothelial adhesion programs	Preclinical stage
**Fusion proteins**	CXCL10‐Fc; CXCL11‐antibody fusions	Extends chemokine half‐life/retention to sustain vascular‐immune crosstalk	Preclinical stage
**Therapeutic Antibodies**	BMS‐986253	Neutralizes CXCL8 to limit myeloid trafficking and restore vascular‐immune balance	NCT03689699, PhaseI/II, Prostate Cancer
**Drugs‐delivery/sequestration**	CXCL10 liposomes; CXCL8‐responsive microspheres; CXCL12‐sequestering NPs	Site‐specific release or chemokine blockade to modulate permeability and T‐cell access	Preclinical stage

### 5.4 Critical Challenges in Clinical Translation of CXC‐targeted Therapies

Despite substantial preclinical evidence, the clinical translation of CXC axis‐targeted therapies has yielded heterogeneous and often inconsistent outcomes. Rather than reflecting insufficient target validation, these outcomes highlight fundamental constraints imposed by the physiological organization of the CXC chemokine network, which complicate direct pharmacological manipulation in clinical settings. This challenge is most clearly illustrated by CXCR4‐targeted strategies. In preclinical models, disruption of the CXCL12‐CXCR4 axis effectively alleviates immune exclusion and modulates vascular‐immune crosstalk. However, in humans, CXCR4 fulfills an essential homeostatic function in hematopoietic stem cell retention, such that sustained pharmacological inhibition induces stem cell mobilization and systemic effects that limit tolerable dose intensity and treatment duration [100]. As a result, CXCR4 inhibition cannot be applied in a prolonged or continuous manner without compromising physiological integrity, thereby constraining the magnitude and persistence of vascular or immune reprogramming achievable in patients.

Importantly, this intrinsic limitation also exposes a second, often underappreciated challenge: the absence of chemokine‐informed patient stratification. Because CXCR4 blockade can only be administered transiently, its therapeutic impact is highly contingent on tumor‐specific reliance on CXCL12‐mediated immune exclusion. Nevertheless, most clinical trials have enrolled broadly inclusive patient populations, despite substantial inter‐tumoral heterogeneity in CXC chemokine program utilization. For example, pancreatic and colorectal cancers frequently exhibit CXCL12‐CXCR4‐dependent stromal sequestration and immune exclusion, whereas immune‐inflamed tumors such as melanoma are predominantly shaped by CXCL9/10‐associated signaling and IFN‐γ‐driven immune infiltration [99, 132, 133, 134]. Applying transient CXCR4 inhibition uniformly across these biologically divergent contexts is therefore unlikely to produce consistent clinical benefit.

Collectively, these observations suggest that inconsistent outcomes are not primarily due to failure of CXC chemokine biology, but are strongly driven by a misalignment between trial design and the context‐ and time‐dependent roles of the CXC network. In this regard, chemokine‐guided patient stratification, identifying tumors in which CXCL12‐CXCR4 signaling is functionally dominant, combined with temporally optimized modulation aligned to functional vascular normalization windows, offers a more realistic translational path than uniform and sustained inhibition of the CXC axis.

## 6 Conclusion and Perspectives

CXC chemokines constitute a pivotal yet incompletely defined bidirectional axis linking angiogenesis, inflammation, and immune remodeling in the TME [15, 134]. Acting as a bidirectional and context‐dependent network, ELR^+^ chemokines such as CXCL1/8 drive endothelial activation and angiogenic sprouting, whereas interferon‐inducible ELR^−^ chemokines including CXCL9/10/11 exert anti‐angiogenic and immune‐activating effects and have been proposed to contribute to vascular functional states that may be compatible with normalization in specific contexts [135, 136]. This functional duality complements and extends the conventional VEGF‐centered paradigm and establishes a rhythmic regulatory system that can be therapeutically harnessed [137, 138]. Furthermore, to augment conventional morphological endpoints, the FVNS integrates chemokine ratios, receptor activity, and immune‐vascular coupling, providing a semi‐quantifiable framework to inform personalized treatment timing.

Translating these functional insights into therapeutic strategies, pharmacologic modulation of the CXC axis has shown significant potential. Targeting CXCR2 or CXCR4 with selective antagonists [139], enhancing the CXCL9/10/11‐CXCR3‐B pathway, or suppressing CXCL8 signaling have been reported to alleviate immune exclusion and improve vascular perfusion, with the potential to prolong normalization‐like windows and enhance synergy between antiangiogenic and immunotherapeutic interventions. Beyond conventional small‐molecule agents, delivery‐based and combinatorial strategies are being developed to achieve stage‐specific and context‐sensitive modulation of CXC‐mediated signaling. Biodegradable release systems and microenvironment‐responsive vectors [140, 141], enable temporally aligned drug delivery that parallels chemokine oscillations, while CXCL9/10/11‐engineered CAR‐T cells [114], CXCR3‐tethered oncolytic viruses [76], and hypoxia‐responsive or immune‐derived nanocarriers [142, 143] demonstrate the potential to synchronize vascular remodeling with immune activation. In addition, biomimetic nanovesicles derived from immune cells and engineered to express CXCR3‐associated motifs while carrying immunostimulatory cytokines such as IL‐12 further refine targeting precision and enhance immune‐cell recruitment toward CXC‐rich vascular niches [144, 145]. Complementing these pharmacologic and delivery advances, computational and AI‐driven approaches, including multi‐omics modeling [146], spatial transcriptomics [147] and deep‐learning‐based compound screening [148], are transforming CXC biology into a programmable and clinically actionable system that bridges molecular regulation with data‐guided therapeutic design.

Despite substantial progress, current understanding of CXC‐mediated vascular reprogramming remains constrained by several challenges. Tumor heterogeneity represents a major limitation, as CXC chemokine expression patterns, ELR^+^/ELR^−^ balance, and immune‐vascular interactions vary markedly across tumor contexts, ranging from CXCL12‐dominated immune‐excluded tumors to IFN‐γ‐associated immune‐inflamed settings [18, 132, 149]. Concurrently, functional redundancy and compensatory signaling within the chemokine network complicate causal attribution of vascular functional outcomes to individual ligands or receptors in vivo [150, 151]. Moreover, the spatiotemporal coordination of ELR^+^ and ELR^−^ chemokine gradients, which has been proposed to influence endothelial behavior and vascular functional transitions, remains technically challenging to resolve, particularly under dynamic in vivo conditions where vascular and immune signals are tightly coupled [152, 153]. Collectively, these constraints indicate that many proposed roles of CXC chemokines in vascular normalization should be interpreted as context‐dependent conceptual models, rather than definitive mechanistic conclusions.

Beyond oncology, the principles of chemokine‐driven vascular reprogramming may extend to chronic inflammation, ischemic injury, and tissue regeneration, where endothelial‐immune dialogue dictates repair or fibrosis outcomes [154, 155]. Understanding this bidirectional and programmable signaling network thus provides a conceptual scaffold for re‐engineering vascular homeostasis across diseases. Altogether, these insights redefine the CXC chemokine network as a programmable vascular‐immune interface, one that not only informs cancer therapy but also offers a conceptual foundation for restoring vascular homeostasis across diverse diseases.

## Conflicts of Interest

The authors declare no conflicts of interest.

## Data Availability

The authors have nothing to report.
